# XRF and XRPD data sets in ternary mixtures with high level micro-absorption and/or preferred orientations problems for phase quantification analysis^☆^^☆☆^

**DOI:** 10.1016/j.dib.2021.107043

**Published:** 2021-04-09

**Authors:** Beatrice Mangolini, Luca Palin, Marco Milanesio, Mattia Lopresti

**Affiliations:** a Universitá del Piemonte Orientale, Dipartimento di Scienze e Innovazione Tecnologica, Viale T. Michel 11, Alessandria 15121, Italy; b Nova Res s.r.l., Via D. Bello 3, Novara 28100, Italy

**Keywords:** X-Ray powder diffraction, Quantitative phase analysis, X-Ray fluorescence, Micro-absorption, Preferred orientation, Polycristalline mixtures, Design of experiment, Simplex-centroid design augmented

## Abstract

Micro-absorption (MA) and/or preferred orientations (PO) are two among the major problems affecting quantitative phase analysis (QPA) by X-ray Powder Diffraction Data (XRPD) in industrial samples such as minerals and ores, additives, cements, friction materials, coal combustion by-products. Typically XRPD data are coupled to elemental analysis by X-ray Fluorescence (XRF) to facilitate phase recognition and quantification when elements heavier than sodium are present. Graphite and urea are typical examples of large production industrial commodities showing such analytical problems. The present article provides a recipe to produce sets of data of increasing difficulty to test the performances of different approaches and/or software’s for QPA by XRPD in graphite, zinc acetate and urea containing samples. Graphite, due to its platelet morphology, can exhibit orientation and was chosen because it is possible to control its PO degree by sieving. Simplex-centroid design augmented was used for the design of the experiments to select the mixtures with the more possible homogeneous exploration of the ternary experimental domains, from pure phase to equal-weighted mixtures. The different data sets collected on the four experimental domains by XRF and XRPD are provided and stored as a repository on Mendeley Data. Using the same approach, additional data sets sets with different composition and/or experimental setup can be added by us or any other contributor with the same DoE approach to create a wide open access data set of standardized X-ray powder diffraction and X-ray fluorescence data.

## Specifications Table

**Table:** 

Subject	Materials science
Specific subject area	Quantitative phase analysis by XRF and XRPD
Type of data	Text files
How data were acquired	X-ray fluorescence
	X-ray powder diffraction
Data format	Raw
Parameters for data collection	XRF and XRPD raw profiles were collected on four different sample groups of mixtures, designed by the simplex-centroid augmented scheme with increasing effects of micro-absorption and/or preferred orientations.
Description of data collection	XRF data were collected in air condition on a Rigaku NEX QC with the X-ray tube at 20 kV and 200 µA. XRPD data were collected by a Bruker D8 Advance in the 2θ range of 10° to 120° with the X-ray tube ad 40 kV electric potential and 40 mA filament current in standard Bragg-Brentano conditions.
Data source location	Institution: Universitá del Piemonte Orientale City/Town/Region: Alessandria Country: Italy
Data accessibility	Repository name: Mendeley Data Data identification number: https://doi.org/10.17632/js2nzwf5md.2 Direct URL to data: https://doi.org/10.17632/js2nzwf5md.2

## Value of the Data

• Chemists, earth and materials scientists, engineers by both industrial and academic fields, interested in solid-state matter analysis can benefit of these data to test and improve the performances and explore the potentialities and limitations of the analytical methods of solid samples. Improving quality control process in commodity production can improve materials usages and reduce wastes and resource consumption. These data will benefit researchers in developing and improving methods, and software and/or analytical approaches can be benchmarked and ranked on the same sets of data to have a clearer view of their accuracy and limitations for quantitative phase analysis in complex samples.
• The data sets available in the literature are natural samples or mixtures prepared with concentrations suitable to reflect real sample compositions [1], [2], [3], [4], [5], [6], [7]. Conversely, the data set D1-4 are the first available XRF/XRPD data sets designed using the DoE theory, therefore by a method-centred approach. A simplex-centroid augmented experimental plan for mixture was used: in this way, the mixture concentrations do not mimic real samples but are optimised to properly sample the experimental domain. This allow the best possible precision in evaluating and comparing methods and/or software used for XRPD/XRF data analysis.
• Methods for phase analysis by X-ray powder diffraction data in presence of preferred orientation and micro-absorption are needed in many industrial applications for the quality control of raw and refined materials. This data set was built measuring samples containing urea and graphite which suffer of such problems and can hardly be quantified with traditional methods of analysis by XRF and XRPD data. The provided data set was built analyzing samples and substances showing different degrees of micro-absorption and preferred orientations, in order to produce standard data set on which different methods can be used for quantification purposes. Patterns and spectra of the pure phases are provided, giving all the necessary tools for both supervised and unsupervised analysis.
• The provided data set is open source and open access and new XRPD/XRF data sets built with the same simplex-centroid design approach but with different samples and/or experimental setup can be added by us or any other contributor. In this way, a wide standardized database of experimental data will be available to carefully test and compare the performances, limitations and possibilities of assessed or new methods of analysis.

## 1 Data Description

**Fig. 1:**
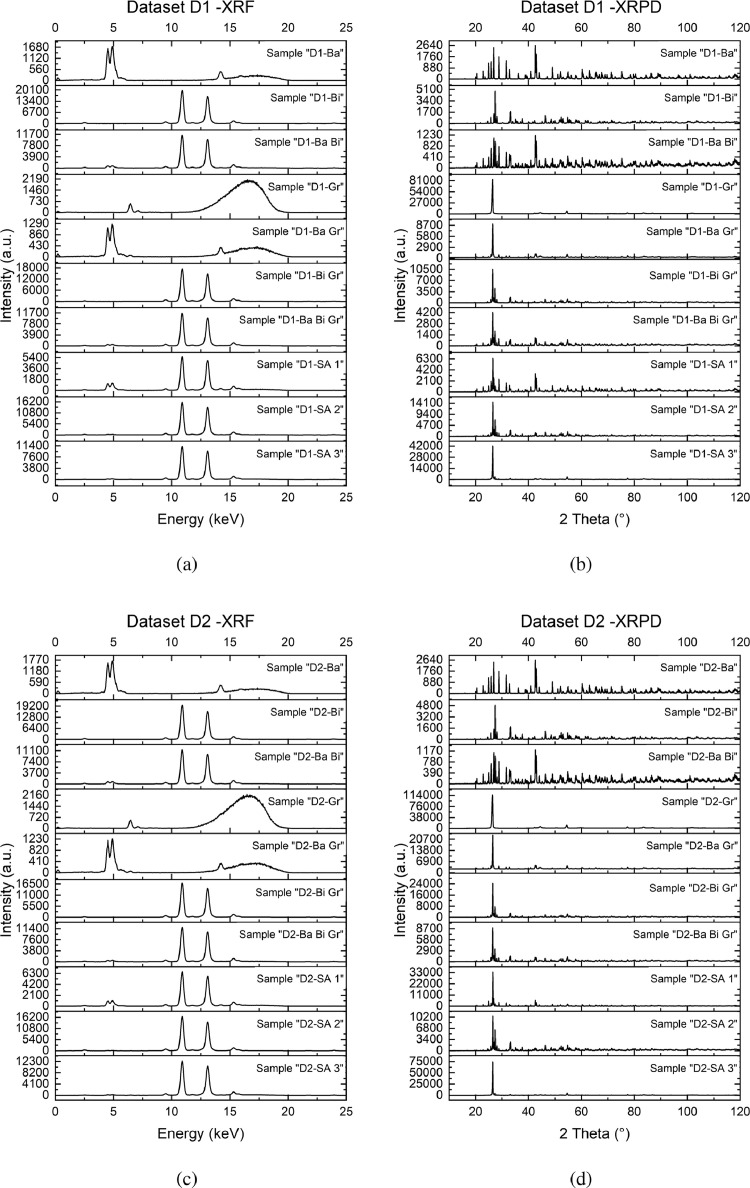
Raw data of experiment set D1 (and) and D2 (and). Figures on the left report XRF data, while figures on the right show XRPD data.

**Fig. 2:**
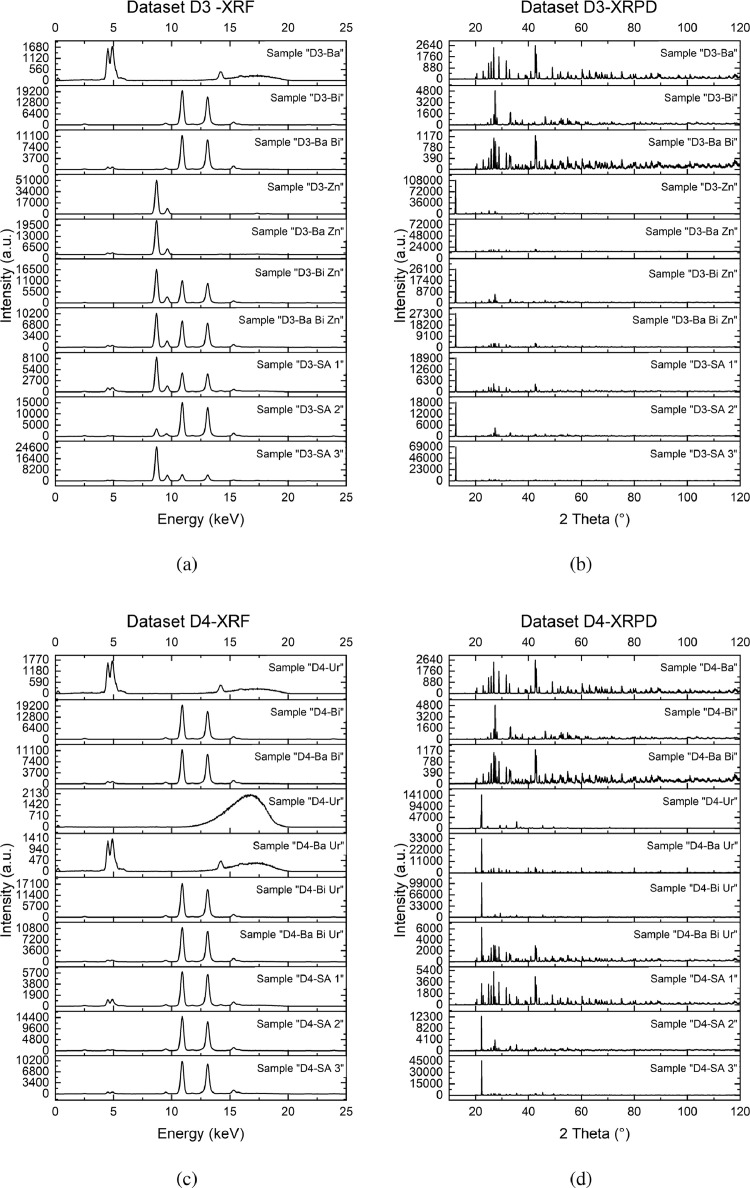
Raw data of experiment set D3 (and) and D4 (and). Figures on the left report XRF data, while figures on the right show XRPD data.

The actual (v.4) version of the repository on Mendeley Data is a collection of 80 raw files organized as described in the following directory tree diagram:



Each level-1 directory (D1, D2, D3 and D4) has the name of the corresponding mixture experimental domain, which is accurately described in the next section. The data set consists in a couple of measurements (one XRF spectrum and one XRPD pattern) collected on each sample of the four sets of experiments. XRF data are provided in two-columns .txt files: intensity (a.u.) vs. energy (keV). XRPD data are instead provided three-column .xye files, in which are reported the intensity (a.u.), the 2θ angles (degrees) and the error associated to the measurement (a.u.). Both XRF and XRPD sets were not pre-processed and are given as obtained by the instruments. Each set of data was collected on mixture samples designed with a simplex-centroid augmented design on four different ternary mixture spaces. The approach, based on DoE, guarantees a full and homogeneous exploration of such experimental domains. The data on experimental set D1 (composed by ${\mathrm{BaSO}}_{4}$, ${\mathrm{Bi}}_{2}O_{3}$ and graphite with diameter lower than 90 µm) are reported in Fig. 1. For readability purposes, XRF data are plotted from 0 keV to 25 keV to better show the principal signals belonging to each pure phase. In the database, data are provided in the full collection range, from 0 keV to 50 keV, although almost no signal is present above 25 keV. As can be seen in Fig. 1, both samples *D1-Ba* and *D1-Bi*, belonging to the pure phases of respectively ${\mathrm{BaSO}}_{4}$ and ${\mathrm{Bi}}_{2}O_{3}$, show the signals of the detectable atoms with this techniques: barium, sulfur and bismuth. However, since the measurement were performed at 20 kV of electric potential (which is the optimal condition for mixtures containing medium and high Z elements) the signal of the sulfur appears very small. The 20 kV measurement condition was chosen to also allow comparison of XRF data with a 20 kV SEM/EDX measurement. SEM/EDX data are not present in this dataset because EDX is not competitive with XRF concerning both analysis duration/complexity and accuracy in quantification, it was used to assess the morphology of mixture components. Sample *D1-Gr*, belonging to graphite, shows Compton scattering only, except for a small signal near 6 keV which was attributed to small impurities of iron that appeared after sieving the powders ($K_{\alpha_{1}\mathrm{Fe}}=6.404\;\text{keV},$ $K_{\alpha_{2}\mathrm{Fe}}=6.391\;\text{keV},$ $K_{\beta_{1}\mathrm{Fe}}=7.057\;\text{keV}$). The physical laws that regulate the signal intensity are not linear combinations of the signal of the pure phases, which is one of the principal obstacles of quantification by XRF. In Fig. diffraction patterns of the same samples are shown. Data were collected in the 2θ range of 10° to 120°, since there were no signals below 10° and peaks belonging to the heavy phases were well visible until very high angles. Samples *D1-Ba* and *D1-Bi* both have patterns very rich in signals with principal peaks intensities in the order of magnitude of $10^{3}$. Those many signals cause peak superposition, resulting in very complex XRPD pattern such as the one in mixture *D1-Ba Bi*. Sample *D1-Gr* shows a very intense peak and few other signals that are about two orders of magnitude lower, which is typical of oriented samples. Effects of micro-absorption can be observed by looking and the differences in the signal intensities: in principle the scattering power increases with increasing Z numbers of the element in each phase. However, the high density of barite and bismite, especially in the case of bismite showing large crystallites, cause and increase absorption of X-ray and net reduced diffraction efficiency. However, barite and bismite both have high densities, which result in crystals where the absorption of the incident X-ray is higher. Data set D2 shows data very similar to data set D1, but in this second collection of data, graphite contains a 30% of large particles (diameters greater than 90 µm) resulting in a much more oriented system with large PO effects. In fact, XRPD data of sample *D2-Gr* show an intense peak which is an order of magnitude greater than the one in sample *D1-Gr*. The effects of preferred orientation and of the micro-absorption are more evident, as the intensities of the peaks in mixtures shows complex features without any clear mathematical relation to those belonging to the pure phases. Data set D3 was collected on an experiment set of mixtures in which graphite was substituted by zinc acetate, therefore a salt with a metallic element which can produce a XRF signal. In Fig. 2(a), in addition to the previously described signals of barite and bismite, the XRF spectrum of zinc acetate can be observed (*D3-Zn*), with two characteristic signals corresponding to its $K_{\alpha }$ and $K_{\beta }$ ($K_{\alpha_{1}\mathrm{Zn}}=8.639\;\text{keV},$ $K_{\alpha_{2}\mathrm{Zn}}=8.616\;\text{keV},$ $K_{\beta_{1}\mathrm{Zn}}=9.572\;\text{keV}$). Zinc acetate, pure and in mixture, is characterized by a principal peak (Fig. 2(b)), 10 to 100 times more intense than the others, which flatten all the other peaks in the pattern. The effect of PO is comparable to the one that affect data set D2. Data set D4 was collected on a ternary mixture experimental domain composed by barium sulfate, bismuth oxide and urea. XRF spectrum of sample *D4-Ur* does not show peaks except for a large band between 14 keV to 19 keV, which is related to the Compton scattering of carbon, nitrogen and oxygen, which do not have an XRF detectable signal. In XRPD data plotted in Fig. 2(d), urea shows a behaviour very similar to zinc acetate and graphite, with a very intense characteristic peak that is two orders of magnitude greater than the most intense peaks of barium sulfate and bismuth oxide.

## 2 Experimental Design, Materials and Methods

### 2.1 Simplex-centroid design augmented

Design of experiments (DoE) is a practice following the “parsimony principle”, consisting in using the smallest number of samples able to give the greatest possible amount of information. This can be done designing experiments homogeneously within the experimental domain, avoiding lined up samples and empty unexplored areas. Most common approaches such as D-optimal, K-optimal, Box-Behnken and Doelhert designs [8] are not suitable for the exploration of a space in which variables cannot be fully independent [9]. Indeed, in mixtures, a fundamental relation states that the sum of every weight fraction $x_{i}$ of all the q components has to be equal to 1, clearly stating the impossibility of the independence principle:

(1) $$\sum_{i=1}^{q}x_{i}=1$$

For this reason, the choice of best DoE approach for mixtures must consider designs appositely made for systems showing such a dependency [9], [10]. Among the many existing solutions (such as simplex-lattice design [11], axial design, ...) simplex-centroid design augmented was chosen because of the low number of experiments required to have a full homogeneous exploration of the mixture space spanning from pure phases to equal-weighted mixtures. Simplex-centroid design is a component-independent experimental design for mixtures in which experiments are generated by the equation:

(2) $$n=2^{q}-1$$

Where n is the number of experiments and q is the maximum number of components of the mixture (corresponding to the dimensionality of the mixture space). While the number of the experiments is defined by Eq. 2, the composition of each mixture is defined by the generic binomial coefficient (qm), where m represents the number of desired components in the mixture. This means:

(3) form=1,(q1)purecomponents,permutationsof(1,0,0,⋯,0)form=2,(q2)mixtures,permutationsof(1/2,1/2,0,⋯,0)form=3,(q3)mixtures,permutationsof(1/3,1/3,1/3,⋯,0)⋯form=q,(qq)mixtureoftheqcomponents(1q,1q,1q,⋯,1q)

**Fig. 3:**
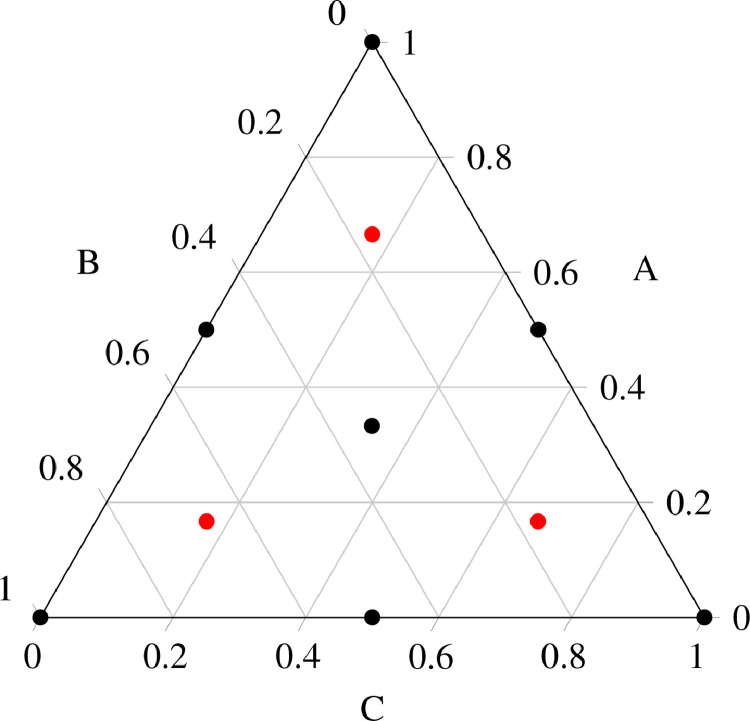
Simplex-centroid design augmented for ternary systems. In black, the $2^{q}+1=7$ points representing the seven mixtures designed from the sole Simplex-Centroid design. In red, the q=3 augmented experiments, for model testing and validation. (For interpretation of the references to colour in this figure legend, the reader is referred to the web version of this article.)

A graphical representation of this experimental design applied to a three-components system, for a generic mixture {A,B,C} can be seen in Fig. 3, represented by the black dots. Pure components are positioned on the vertices of an equilateral triangle, two-component mixtures are positioned in the midpoint of each side and the ternary mixture is positioned in the center of gravity of the triangle. The red points in the ternary graph represent the “augmented” experiments of the simplex-centroid design [9], which is a set of additional experiments which give the possibility to obtain more precise information on the curvature of the response surface inside the experimental domain. With an equal number of experiments, another DoE (such as simplex-lattice design {3,2} [11]) would give more information about the perimeter of the experimental domain, neglecting the central zone of the mixture space. This set of experiments is commonly used for model validation and testing, but can also be used to build more robust models. The set of augmented experiments is obtained by the q permutations of the $\left(\frac{q+1}{2q},\frac{1}{2q},\frac{1}{2q},\cdots ,\frac{1}{2q}\right)$ mixture, which results in three additional experiment for a three-component mixture domain.

### 2.2 Materials

Blanc Fixe G barium sulfate (>99% w/w, MM = 137.327 g mol^−1^, ρ=4.48 g cm^−3^) were given by Universal Services S.r.l. (Milano, Italy) and produced by Solvay (Massa, Italy). ${\mathrm{Bi}}_{2}O_{3}$ (>99% w/w, MM = 208.980 g mol^−1^, ρ=8.93 g cm^−3^) was purchased by Thermo Fischer (Kandel, Germany). Zinc acetate (>99% w/w, MM = 219.528 g mol^−1^, ρ=1.74 g cm^−3^) and urea (>99% w/w, MM = 60.06 g mol^−1^, ρ=1.32 g cm^−3^) were purchased by Merck KGaA (Darmstadt, Germany). Graphite (>99% w/w, MM = 12.01 g mol^−1^ ρ=2.23 g cm^−3^) was given by Itaprochim (Milan, Italy).

### 2.3 Data collection

A Rigaku NEX QC with the X-ray tube at 20 kV in electric potential and 200 µA in current was used for XRF measurement in air condition. Rigaku’s filter A was used during the measurements. A Bruker D8 Advance with a Lynxeye XE-T detector and a Cu source (λ= 1.54 Å) was used for XRPD pattern collection in the 2θ range of 10° to 120°). The instrument was equipped with an auto-sampler with nine positions and an air scatter knife. On both the primary and secondary optics Soller slits 2.5° opening were positioned and automatic divergence slits were used with a fixed sample illumination of 10 mm length. Goniometer radius was 280 mm. Analyses were run without sample rotation as in preliminary measurements it was observed to negatively affect the quality of data. In fact, the large differences in density cause a segregation during rotation because heavy and light phases are concentrated in the bottom and top parts of the sample holder respectively. Such phenomenon could be avoided by pressing the sample in a pellet but this would cause an increased and not controllable degree of PO. The tube was set at 40 mA in current and 40 kV in electric potential. Powders were observed with a STEMI 508 microscope with a 2x frontal optic equipped with Zeiss fiber optics halogen bulbs and a LED ring for illumination. Digital high resolution images of the powders were collected with a 20 MPx SONY sensor camera. A Hitachi FLEXSEM 1000 equipped with AZtecOne Oxford EDS, was used for electron microscopy analysis and elemental analysis, in order to evaluate samples’ features. The source of the electrons was a tungsten filament at 20 kV. Powders were measured without coatings.

### 2.4 Sample preparation

**Fig. 4:**
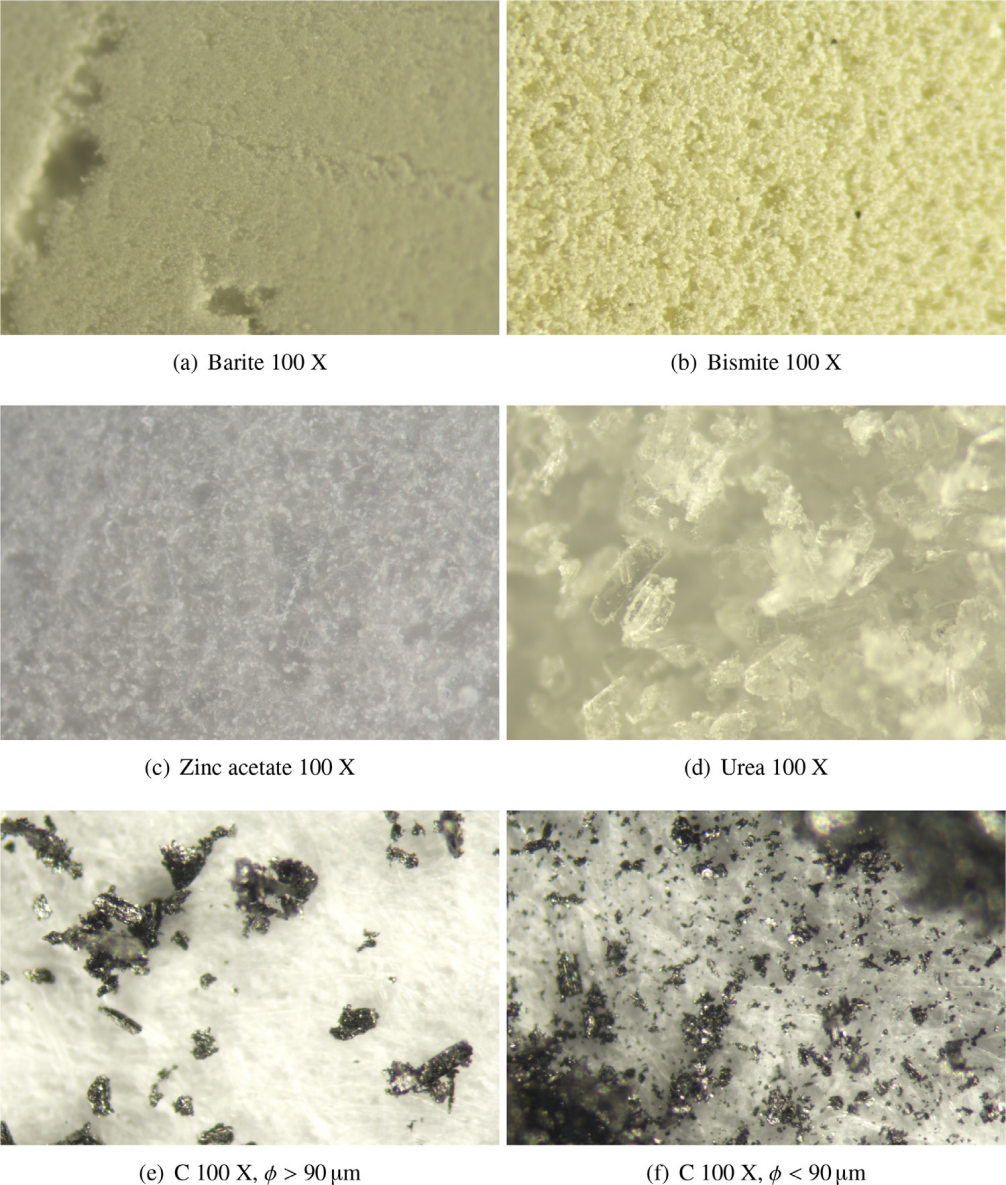
Optical microscope images of pure phases used in all the experimental designs. Particle size are very different from one substance to another promoting micro-absorption effects. Graphite is divided in two batches due to sieving, as shown in figures.

Substances were only manually ground prior measurement in an agate mortar to simulate an industrial content in which simple and fast measurements are preferred (i.e. avoid Mc Crone grinding prior XRPD measurement). Graphite, the compound showing layered morphology and thus more prone to PO, was then sieved (90 µm mesh diameter) and both the finer and the larger fractions were retained with the scope of preparing samples with different but controlled presence of PO. Images of pure phases, collected with a STEMI 508 microscope with a 2x frontal optic, are shown in Fig. 4. 4.5 g of each sample were prepared. The quantity was chosen accordingly to the UNI EN 15309:2007 Italian standard methodology for the determination of elemental by X-ray fluorescence [12] as there are no particular requirements for XRPD.

Mixtures sets, whose compositions are detailed in Table 1, were prepared according to a Simplex-centroid design augmented and characterized by the following: four sets of samples were then produced, in order to simulate an equal number of different systems with different typical obstacles for the quantitative phase analysis: complexity of the mixture by the increasing peak superposition, preferred orientations and high differences in density and particle size of the phases are also present, to enhance the effects of micro-absorption. The 4 data sets have been built with the following features:

• Experiments set D1: the three components are barite, bismite and the sieved graphite (ϕ<90 µm), showing a limited PO effect. The main issues related to this data set are the large differences in density and in the particle size of the three pure phases and the presence of a phase (graphite) which does not have a characteristic XRF signal.
• Experiments set D2: identical to experimental set D1, but 30% in weight of the used graphite belonged the fraction with diameter larger than 90 µm after sieving. As a result, an important PO effect in addition to the previously described issues of experiment set D1 is introduced in experiment set D2.
• Experiments set D3: graphite is substituted by zinc acetate, which possesses an XRF signal ($K_{\alpha \mathrm{Zn}}=8.63\;\text{keV},$ $K_{\beta \mathrm{Zn}}=9.57\;\text{keV}$), shows PO and has a lower density, which enhances the micro-absorption effects and a larger unit cell, increasing peak superposition effects.
• Experiments set D4: in this set, the third component is urea, which once again does not possesses a XRF signal, has a slight PO effect, has larger particles (as can be seen in Fig. 4) and has a lower density than zinc acetate, with increased with in micro-absorption effects due to density difference with respect to barite and bismite.

**Table 1:** Compositions and ID of every produced sample. The four experimental design are ordered in an increasing degree of influence of the micro-absorption and PO effect. Graphite quantities in experiments set D2 are reported as total weight fraction outside the brackets and in relative quantities of the larger and smaller fractions inside the brackets.

	BaSO${}_{4}$	Bi${}_{2}$O${}_{3}$	Zn(CH${}_{3}$COO)${}_{2}$(H${}_{2}$O)${}_{2}$	CH${}_{2}$N${}_{2}$O	C ${\left({\text{C}}_{\text{or.}}-{\text{C}}_{\text{n.or.}}\right)}^{a}$
Sample ID	(% w/w)	(% w/w)	(% w/w)	(% w/w)	(% w/w)
Experiments set D1					
D1-Ba	1.000	0.000	-	-	0.000 (0-0)
D1-Bi	0.000	1.000	-	-	0.000 (0-0)
D1-Gr	0.000	0.000	-	-	1.000 (0–1.000)
D1-Ba Bi	0.500	0.500	-	-	0.000 (0-0)
D1-Ba Gr	0.500	0.000	-	-	0.500 (0-0.500)
D1-Bi Gr	0.000	0.500	-	-	0.500 (0-0.500)
D1-Ba Bi Gr	0.333	0.333	-	-	0.333 (0-0.333)
D1-SA 1	0.666	0.167	-	-	0.167 (0-0.167)
D1-SA 2	0.167	0.666	-	-	0.167 (0-0.167)
D1-SA 3	0.167	0.167	-	-	0.666 (0-0.666)

Experiments set D2					
D2-Ba	1.000	0.000	-	-	0.000 (0-0)
D2-Bi	0.000	1.000	-	-	0.000 (0-0)
D2-Gr	0.000	0.000	-	-	1.000 (0.310-0.690)
D2-Ba Bi	0.500	0.500	-	-	0.000 (0-0)
D2-Ba Gr	0.500	0.000	-	-	0.500 (0.155-0.345)
D2-Bi Gr	0.000	0.500	-	-	0.500 (0.155-0.345)
D2-Ba Bi Gr	0.333	0.333	-	-	0.333 (0.103-0.230)
D2-SA 1	0.666	0.167	-	-	0.167 (0.052-0.115)
D2-SA 2	0.167	0.666	-	-	0.167 (0.052-0.115)
D2-SA 3	0.167	0.167	-	-	0.666 (0.206-0.460)

Experiments set D3					
D3-Ba	1.000	0.000	0.000	-	-
D3-Bi	0.000	1.000	0.000	-	-
D3-Zn	0.000	0.000	1.000	-	-
D3-Ba Bi	0.500	0.500	0.000	-	-
D3-Ba Zn	0.500	0.000	0.500	-	-
D3-Bi Zn	0.000	0.500	0.500	-	-
D3-Ba Bi Zn	0.333	0.333	0.333	-	-
D3-SA 1	0.666	0.167	0.167	-	-
D3-SA 2	0.167	0.666	0.167	-	-
D3-SA 3	0.167	0.167	0.666	-	-

Experiments set D4					
D4-Ba	1.000	0.000	-	0.000	-
D4-Bi	0.000	1.000	-	0.000	-
D4-Ur	0.000	0.000	-	1.000	-
D4-Ba Bi	0.500	0.500	-	0.000	-
D4-Ba Ur	0.500	0.000	-	0.500	-
D4-Bi Ur	0.000	0.500	-	0.500	-
D4-Ba Bi Ur	0.333	0.333	-	0.333	-
D4-SA 1	0.666	0.167	-	0.167	-
D4-SA 2	0.167	0.666	-	0.167	-
D4-SA 3	0.167	0.167	-	0.666	-

${}^{a}$Quantities of oriented graphite (${\text{C}}_{\text{or.}},\phi >90\;\mu \text{m}$) and non-oriented graphite $\left({\text{C}}_{\text{n.or.}},\phi <90\;\mu \text{m}\right)$.

**Fig. 5:**
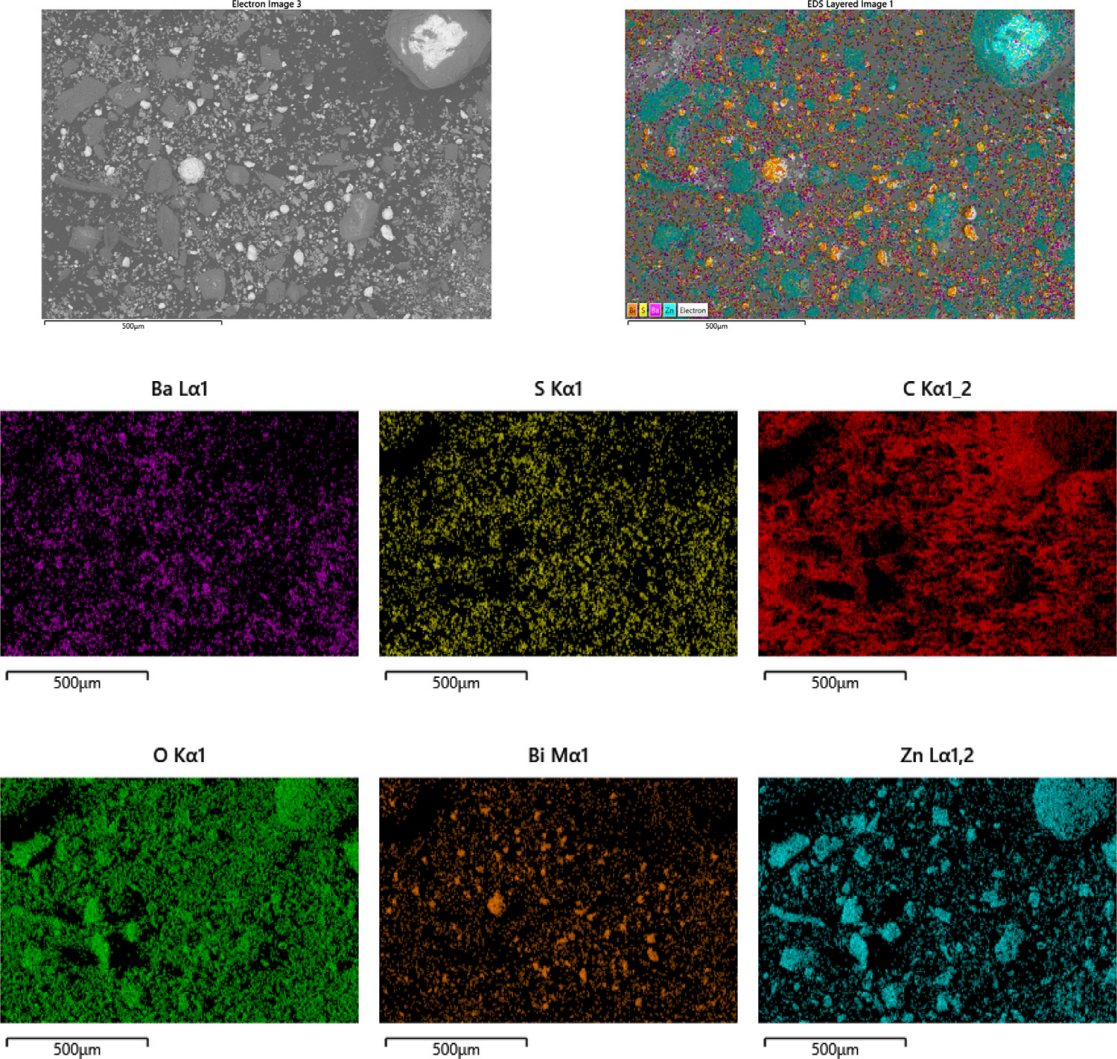
SEM-EDS images of sample *D3-Ba Bi Zn*. Although being accurately dispersed, large differences in particle size of each powder is clearly visible. A large agglomerate of zinc acetate can be observed in the top-right area of the pictures.

SEM-EDS analysis was used for electron microscopy analysis and elemental analysis, in order to evaluate samples’ correct dispersion and differences in particle size. In this paper, an example of the distribution of the mixtures prepared is reported in Fig. 5, where the dispersion of the three phases (barium sulfate, bismuth oxide and zinc acetate) can be observed. Although being accurately mixed, the differences in the average particle size of the pure substances affect the homogeneity of the powder mixture. In particular, a large agglomerate of almost 300 µm can be observed on the top-right corner of the figures.

## Open Database Rules

Any one can add a XRPD data set provided that:

• XRPD data and instrumental conditions are provided to the corresponding author to produce a new updated version of the Mendeley Data repository;
• the simplex-centroid augmented approach must be used to produce ten XRPD patterns and, if possible, the ten corresponding XRF spectra for each proposed dataset;
• all the instrumental data set to both reproduce the experiments and using parametric approach for data refinement and/or analysis must be provided.

## Authors’ Contribution

Mixtures were prepared by BM. XRF and XRPD analysis was performed by BM. Materials characterization by optical microscopy, SEM and EDS was performed by both BM and LP. All the authors analyzed the data, edited the manuscript and approved its final version.

## Declaration of Competing Interest

The authors declare that they have no known competing financial interests or personal relationships which have, or could be perceived to have, influenced the work reported in this article.
